# Survival benefits of postoperative radiotherapy in patients with cT_1 − 2_N_1_M_0_ breast cancer after neoadjuvant chemotherapy: a SEER-based population study

**DOI:** 10.1186/s12905-024-03165-1

**Published:** 2024-06-05

**Authors:** Jie Yang, Jie Zhao, Hui Chang, Lijuan Yan, Jinru Zhang, Haiming Liu, Peng Ning

**Affiliations:** Department of Oncology, Baoji Gaoxin Hospital, No.19, Gaoxin 4 Road, Gaoxin District, Baoji, Shaanxi Province 721000 China

**Keywords:** cT_1 − 2_N_1_M_0_ breast cancer, Neoadjuvant chemotherapy, Postoperative radiotherapy, SEER, Prognosis

## Abstract

**Background:**

Whether patients with cT_1 − 2_N_1_M_0_ breast cancer can benefit from postoperative radiotherapy (RT) after receiving neoadjuvant chemotherapy (NAC) has been controversial. Therefore, the purpose of this study was to explore whether postoperative RT can benefit this group of patients in terms of survival.

**Methods:**

We used Surveillance, Epidemiology, and End Results (SEER) data to conduct a retrospective review of women with cT_1 − 2_N_1_M_0_ breast cancer diagnosed between 20 and 80 years of age who received NAC between 2010 and 2015. Our study compared the impact of postoperative RT on overall survival (OS) and cancer-specific survival (CSS) in breast cancer patients using propensity score matching (PSM) and performed subgroup analysis.

**Results:**

This study finally included 1092 cT_1 − 2_N_1_M_0_ breast cancer patients. Regardless of the patient’s PSM status, postoperative RT was significantly associated with OS of cT_1-__2_N_1_M_0_ breast cancer patients who received NAC. Specifically, the 10-year OS rate was 78.7% before PSM matching, compared with 71.1% in patients who did not receive postoperative RT, and the difference was more significant after PSM matching, which was 83.1% and 71.1% respectively. However, postoperative RT did not significantly benefit CSS in patients with cT_1 − 2_N_1_M_0_ breast cancer who received NAC. The 10-year CSS rate was 81.4% VS 76.2% (*P* = 0.085) before PSM matching and 85.8% VS 76.2%(*P* = 0.076) after matching. Due to the intersection of OS and CSS curves, this restricted mean survival time (RMST) method was chosen as a supplement. After 60 months, the OS difference in RMST between the postoperative RT group and the non-radiotherapy (noRT) group was 7.37 months (95%CI: 0.54–14.21; *P* = 0.034), and the CSS difference was 5.18 months (95%CI: -1.31-11.68; *P* = 0.118). Subgroup analysis found that in patients with right-sided breast cancer, postoperative RT improved the patient’s OS (HR = 0.45, 95%CI: 0.21–0.95, *P* = 0.037) and CSS (HR = 0.42, 95%CI: 0.18–0.98, *P* = 0.045).

**Conclusions:**

Our results showed that additional postoperative RT improved the OS of cT_1 − 2_N_1_M_0_ breast cancer patients who received NAC, but failed to improve their CSS. It is worth noting that in the subgroup analysis of patients with right-sided breast cancer, we observed significant improvements in OS and CSS. And further prospective studies are still needed to verify the effect of postoperative RT in different subgroups.

## Introduction

Breast cancer is not only the most common malignancy in women but also one of the leading causes of cancer-related death [1]. Neoadjuvant chemotherapy (NAC) was initially used to treat patients with locally advanced breast cancer, but its use has expanded to early-stage breast cancer, especially those with high-risk early-stage breast cancer, such as triple-negative, HER2-positive, and axillary node-positive of breast cancer patients [2–4]. NAC has been proven to improve the pathological complete response rate (pCR) of breast cancer patients, with no significant difference in overall survival (OS) compared with postoperative chemotherapy [5, 6]. In addition, a meta-analysis including 10 randomized controlled trials also confirmed that there was no significant difference in distant recurrence rate and cancer-specific survival (CSS) between these two treatment strategies [7] At the same time, compared with postoperative chemotherapy, NAC has shown obvious advantages in increasing the rate of breast-conserving surgery (BCS), guiding the selection of subsequent treatment options, and improving patients’ compliance with treatment [8]. However, with the wide application of NAC in clinical practice, the indications of postoperative radiotherapy (RT) for breast cancer patients after NAC are facing challenges, because the existing guidelines for breast cancer RT are mainly based on the results of breast cancer patients treated by direct surgery, rather than breast cancer patients receiving NAC.

It is generally believed that patients with initial stage III breast cancer treated with NAC can benefit from postoperative RT, while patients with cT_1-2_N_0_M_0_ are unlikely to benefit from postoperative RT. For patients with cT_1-2_N_1_M_0_ breast cancer, it is still unclear whether postoperative RT can bring benefits. Recently, a prospective randomized controlled RAPCHEM study [9] included 838 cT_1 − 2_N_1_M_0_ breast cancer patients who received NAC and were grouped according to the number of postoperative lymph node positives. The study found that the 5-year local recurrence rates (LRR) of the ypN_0_ group that did not receive RT, the ypN_1_ group and ypN_2-3_ group that received RT were 2.1% (95%CI:0.9–4.3), 2.2% (95%CI:1.0-4.1), and 2.3% (95%CI:0.8–5.5), respectively. Therefore, it is considered that it is safe to waive RT for ypN_0_ patients in this population.

However, so far, it remains unclear whether cT_1 − 2_N_1_M_0_ breast cancer patients receiving NAC derive survival benefit from postoperative RT. Therefore, in this retrospective analysis study based on the Surveillance, Epidemiology, and End Results database (SEER), we aimed to evaluate the survival benefit of postoperative RT in patients with cT_1 − 2_N_1_M_0_ breast cancer who received NAC. In addition, we applied the propensity score matching (PSM) analysis method, which aims to reduce the influence of potential confounding factors in non-randomized controlled studies. At the same time, through subgroup analysis of breast cancer patients, we further explored which specific groups of breast cancer patients would be more beneficial to postoperative RT.

## Methods

### Data source

This study is a population study based on a publicly available SEER database. Therefore, our study did not require ethical approval and had the advantages of large sample size and guaranteed data quality. We obtained permission to access SEER Research Plus Data, 2021 Sub (1975–2019) with reference number 18,430-Nov 2021.

The study subjects were initially screened as patients aged 20 to 80 years old who were diagnosed with a single primary breast malignant tumor between 2010 and 2015. We collected clinicopathological variables including diagnosis year, age, race, marital status, tumor laterality, specific tumor location, histological type, tumor grade, T stage, N stage, M stage, type of surgery, number of postoperative positive lymph nodes, breast subtype, whether to receive RT, whether to receive chemotherapy, survival status, cause of death and survival time. Definition: NAC is systemic therapy before surgery, and RT is defined as RT after surgery, which only includes external beam RT.

**Fig. 1:**
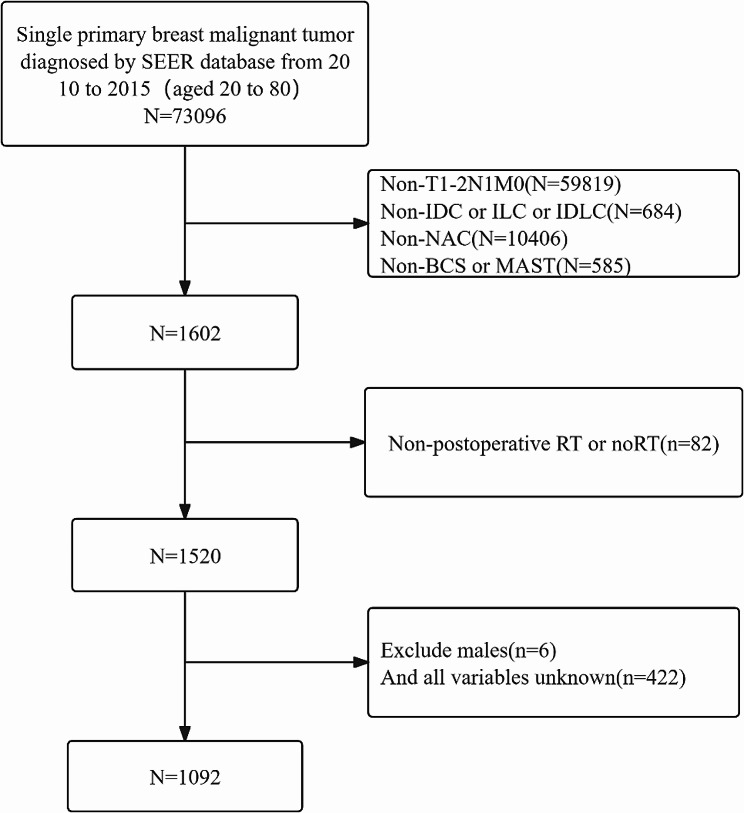
Flow chart of inclusion and exclusion of breast cancer patients from 2010 to 2015. Abbreviations: RT, radiotherapy; noRT, non-radiotherapy; IDC, invasive ductal carcinoma; ILC, invasive lobular carcinoma; IDLC, invasive ductal and lobular carcinoma; BCS, breast-conserving surgery; MAST, mastectomy

To ensure the accuracy and reliability of our study, we set strict inclusion and exclusion criteria. Inclusion criteria include: (1) The tumor is stage T_1 − 2_N_1_M_0_; (2) The pathological type is invasive ductal carcinoma (IDC), invasive lobular carcinoma (ILC) or mixed invasive ductal and lobular carcinoma (IDLC); (3) All patients All received NAC; (4) The types of surgery were limited to BCS and mastectomy (MAST); (5) Postoperative RT or non-radiotherapy(noRT). Exclusion criteria include: (1) Male patients; (2) Records containing any unknown variables in the data (as shown in Fig. 1).

### Statistical analysis

The chi-square test was used to compare the differences in clinical and pathological characteristics between the patient groups who received and did not receive RT. PSM was employed to balance the two groups of clinical and pathological features using a 1:1 ratio, nearest neighbor matching, and a caliper of 0.2.The Kaplan-Meier method was used to estimate OS and CSS, and the log-rank test was used to compare survival differences. When the proportional hazard hypothesis is not satisfied, the restrictive mean survival time (RMST) is used for supplementary analysis. Statistical analysis was done by R software (4.0.3), *P* < 0.05 was considered to be statistically significant.

## Results

### Patient characteristics

Between 2010 and 2015, the SEER database recorded 1,092 patients with cT_1 − 2_N_1_M_0_ stage breast cancer who received NAC followed by surgical treatment, including BCS and MAST. Of these patients, 900 received postoperative RT, while 192 did not receive postoperative RT. The vast majority of patients are aged between 40 and 59 years old (59.1%), white (70.3%) and married (61.8%). The most common histological type is IDC, accounting for 93.1%. It is worth mentioning that among patients who received postoperative RT, a higher proportion of patients chose BCS. There was no significant difference in age, race, year of diagnosis, histological type, pathological grade, lateral tumor location, cT stage and the number of postoperative positive lymph nodes between the two groups. In order to balance the differences in surgical methods between the two groups, the PSM method was used, and the final matching sample included 192 patients who received postoperative RT and 192 patients who did not receive postoperative RT. The baseline clinicopathological characteristics of the patient groups before and after PSM are detailed in Table 1.

**Table 1:** Baseline characteristics of patients included in the analysis before and after PSM

Characteristic	Before PSM			After PSM		
	noRT (*N*, %)	RT (*N*, %)	*P*	noRT (*N*, %)	RT (*N*, %)	*P*
**Year of diagnosis**			0.074			0.052
2010–2012	93 (48.4)	370 (41.1)		93 (48.4)	113 (58.9)	
2013–2015	99 (51.6)	530 (58.9)		99 (51.6)	79 (41.1)	
**Age**			0.331			0.786
20–39 years	23 (12.0)	145 (16.1)		23 (12.0)	23 (12.0)	
40–59 years	116 (60.4)	529 (58.8)		116 (60.4)	110 (57.3)	
60–79 years	53 (27.6)	226 (25.1)		53 (27.6)	59 (30.7)	
**Marital**			0.847			0.752
married	117 (60.9)	558 (62.0)		117 (60.9)	121 (63.0)	
nomarried	75 (39.1)	342 (38.0)		75 (39.1)	71 (37.0)	
**Race**			0.260			0.427
Black and Other	50 (26.0)	274 (30.4)		50 (26.0)	58 (30.2)	
White	142 (74.0)	626 (69.6)		142 (74.0)	134 (69.8)	
**Laterality**			0.150			1.00
Left	111 (57.8)	466 (51.8)		111 (57.8)	111 (57.8)	
Right	81 (42.2)	434 (48.2)		81 (42.2)	81 (42.2)	
**Location**			0.090			0.648
Upper/Lower-inner quadrant	29 (15.1)	133 (14.8)		29 (15.1)	34 (17.7)	
Upper/Lower-outer quadrant	81 (42.2)	453 (50.3)		81 (42.2)	73 (38.0)	
other	82 (42.7)	314 (34.9)		82 (42.7)	85 (44.3)	
**T stage**			0.501			0.724
T1	50 (26.0)	211 (23.4)		50 (26.0)	46 (24.0)	
T2	142 (74.0)	689 (76.6)		142 (74.0)	146 (76.0)	
**Histology**			0.958			0.747
IDC	178 (92.7)	839 (93.2)		178 (92.7%)	181 (94.3)	
ILC	5 (2.6)	23 (2.6)		5 (2.6)	3 (1.6)	
IDLC	9 (4.7)	38 (4.2)		9 (4.7)	8 (4.2)	
**Grade**			0.489			0.860
Well differentiated	11 (5.7)	55 (6.1)		11 (5.7)	11 (5.7)	
Moderately differentiated	62 (32.3)	329 (36.6)		62 (32.3)	67 (34.9)	
Poorly differentiated	119 (62.0)	516 (57.3)		119 (62.0)	114 (59.4)	
**Breast surgery**			< 0.001			0.396
BCS	65 (33.9)	537 (59.7)		65 (33.9)	74 (38.5)	
MAST	127 (66.1)	363 (40.3)		127 (66.1)	118 (61.5)	
**Positive lymph nodes**			0.741			0.570
0	36 (18.8)	151 (16.8)		36 (18.8)	39 (20.3)	
1∼3	146 (76.0)	694 (77.1)		146 (76.0)	147 (76.6)	
≥4	10 (5.2)	55 (6.1)		10 (5.2)	6 (3.1)	
**Breast Subtype**			0.468			0.860
HR-/HER2-	43 (22.4)	180 (20.0)		43 (22.4)	41 (21.4)	
HR-/HER2+	22 (11.5)	78 (8.7)		22 (11.5)	27 (14.1)	
HR+/HER2-	83 (43.2)	431 (47.9)		83 (43.2)	78 (40.6)	
HR+/HER2+	44 (22.9)	211 (23.4)		44 (22.9)	46 (24.0)	

Abbreviations: RT, radiotherapy; noRT, non-radiotherapy; IDC, invasive ductal carcinoma; ILC, invasive lobular carcinoma; IDLC, invasive ductal and lobular carcinoma; BCS, breast-conserving surgery; MAST, mastectomy

### Survival before PSM

**Fig. 2:**
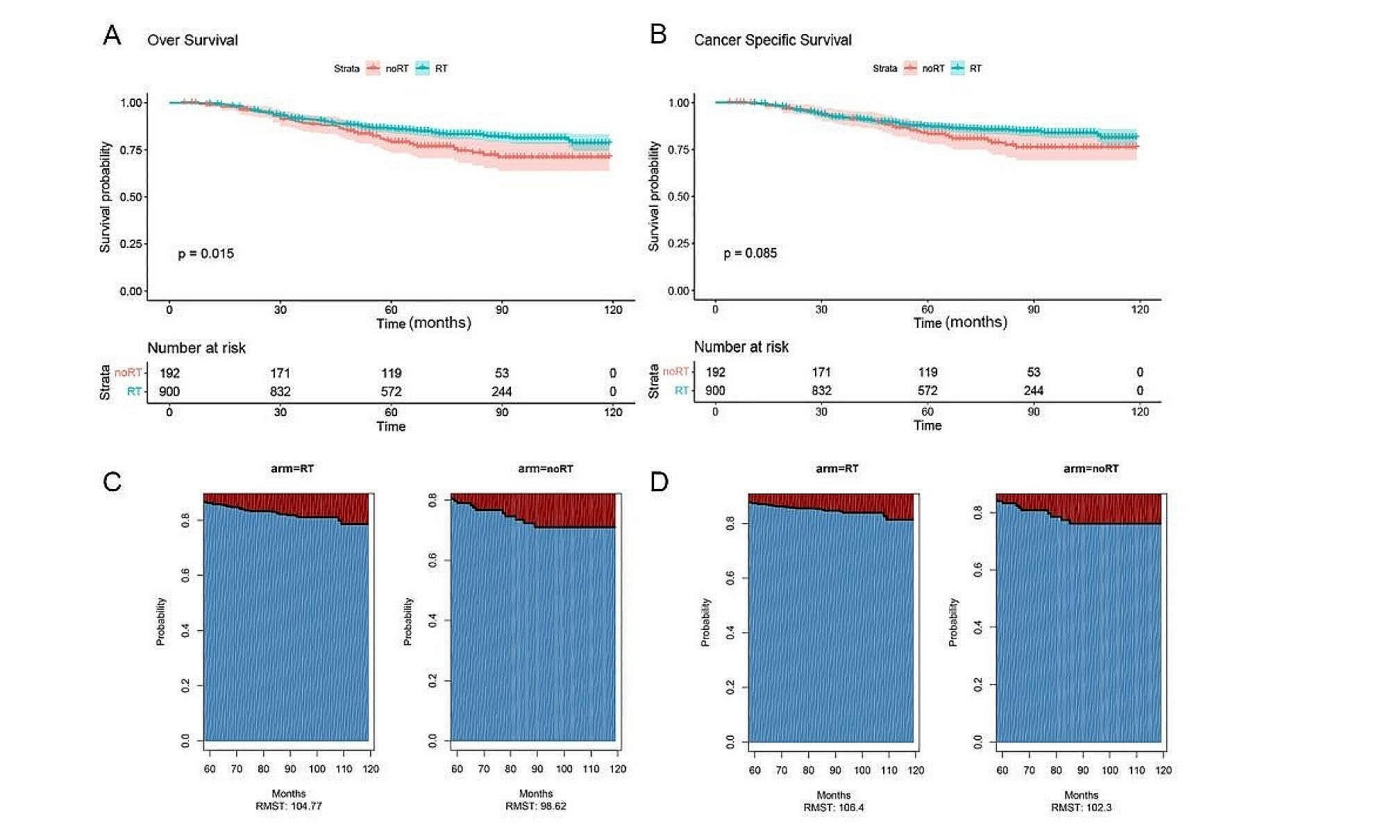
Kaplan-Meier OS (**A**) and CSS (**B**) curves by RT status before PSM, and comparison of average OS (**C**) and CSS (**D**) survival time between RT and noRT groups using RMST before PSM

In this study, the median follow-up time for eligible patients was 69.0 months (IQR: 52, 91 months). The Kaplan-Meier survival curve (Fig. 2A and B) showed that patients who received RT had significant benefits in terms of OS compared with those who did not receive RT (*P* = 0.015). The 10-year OS rate was 78.7% (95% CI: 74.3-83.3%) in the RT group and 71.1% (95% CI: 63.8-79.1%) in the noRT group. However, the difference in CSS between the two groups did not reach statistical significance (*P* = 0.085). The 10-year CSS rate was slightly higher in the RT group, 81.4% (95% CI:77.1-85.9%), and 76.2% (95% CI:69.2-83.8%) in the noRT group. Given that the Kaplan-Meier curves for OS and CSS crossed, which indicated that the assumption of equal risks was not tenable, RMST was used for analysis. Figure 2C and D show that after 60 months, the OS difference of RMST of patients who received RT compared with those who did not receive RT was 6.15 months (95% CI: 0.52–11.78, *P* = 0.032), and the difference of CSS was 4.10 months (95% CI: -1.21-9.41, *P* = 0.130).

### Survival after PSM

**Fig. 3:**
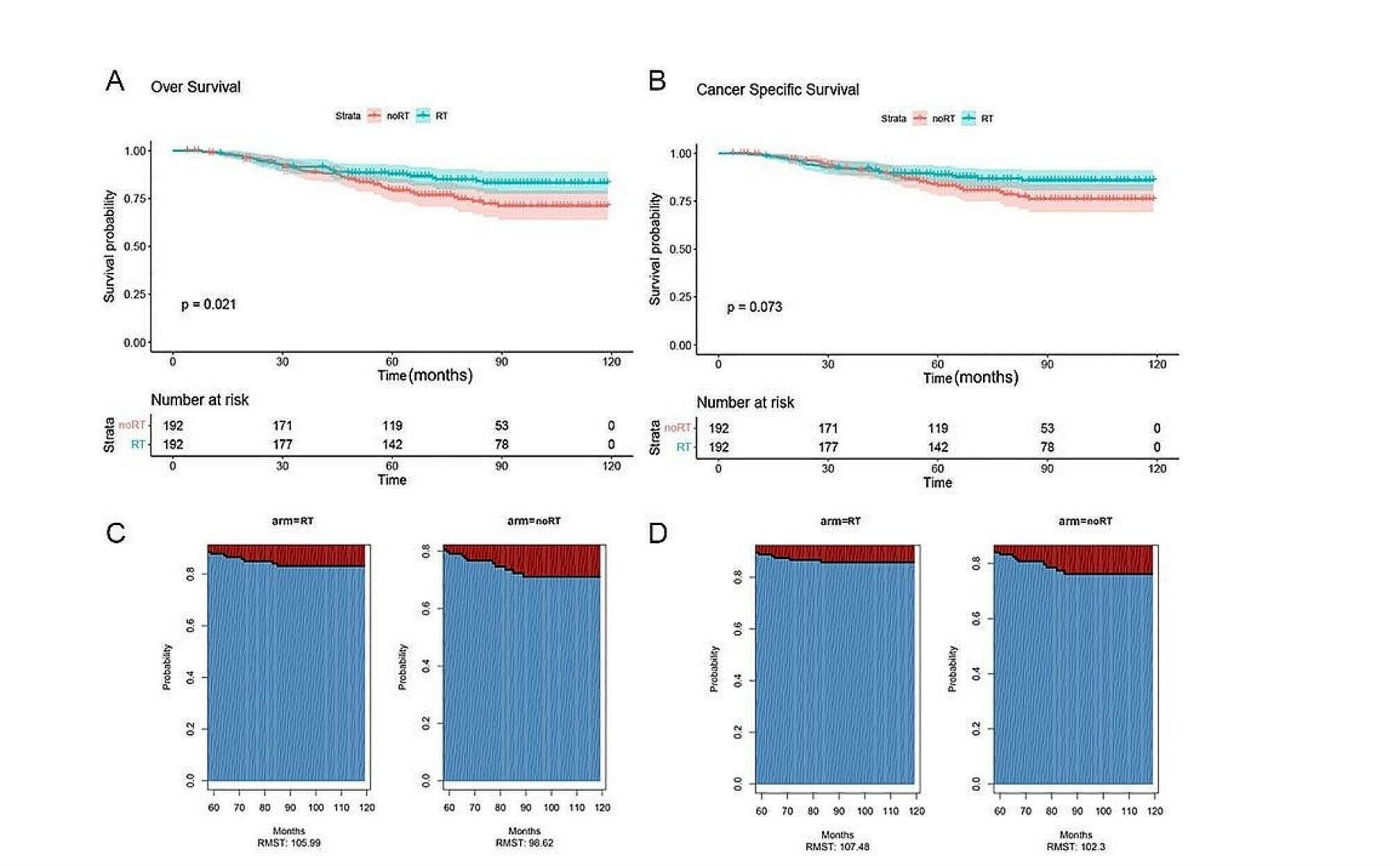
Kaplan-Meier OS (**A**) and CSS (**B**) curves by RT status after PSM, and comparison of average OS (**C**) and CSS (**D**) survival time between RT and noRT groups using RMST after PSM

After PSM, compared with the group not receiving RT, the 10-year OS rate of the RT group still showed a significant advantage (*P* = 0.021) (Fig. 3A), and the RT group was 83.1% (95%CI: 77.6-89.1%), the rate in the group that did not receive RT was 71.1% (95% CI: 63.8-79.1%). Although the 10-year CSS rate in the RT group was higher than that in the group not receiving RT (85.8% (95%CI: 80.7-91.2%) VS 76.2% (95%CI: 69.2-83.8%)), this difference was not statistically significant (*P* = 0.073) (Fig. 3B). Similar to before PSM, the OS and CSS curves also cross, and RMST is used for analysis. After 60 months, the OS difference in RMST between the postoperative RT group and the noRT group was 7.37 months (95% CI: 0.54–14.21, *P* = 0.034) (Fig. 3C), and the CSS difference was 5.18 months (95% CI: -1.31-11.68, *P* = 0.118) (Fig. 3D).

**Fig. 4:**
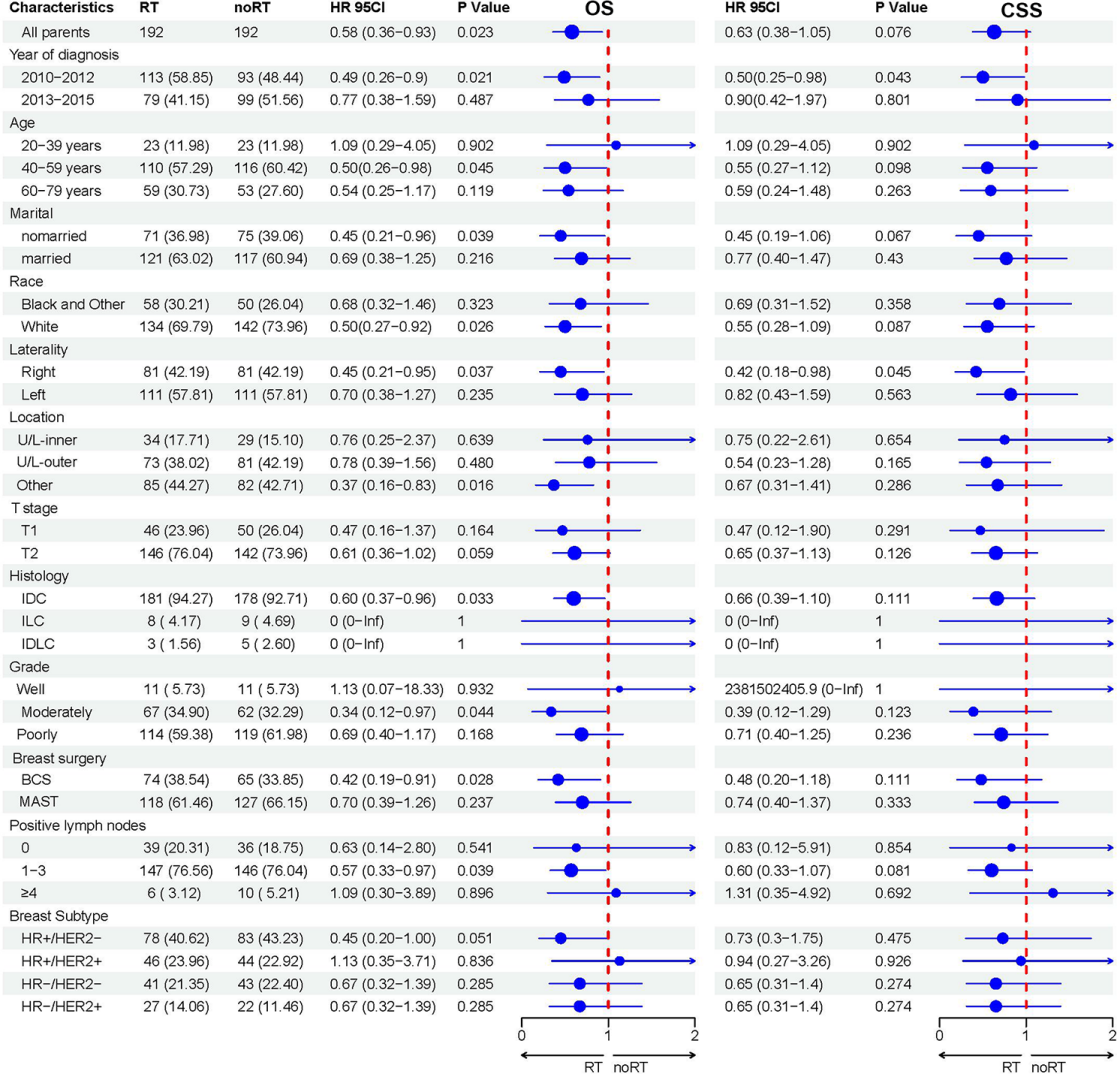
Forest map for subgroup analysis of the effects of RT status on OS and CSS after PSM. Abbreviations: RT, radiotherapy; noRT, non-radiotherapy; IDC, invasive ductal carcinoma; ILC, invasive lobular carcinoma; IDLC, invasive ductal and lobular carcinoma; BCS, breast-conserving surgery; MAST, mastectomy

In addition, according to the subgroup analysis results after PSM (Fig. 4), both OS and CSS showed significant improvement in the subgroup of patients with right-sided breast cancer. However, only a significant increase in OS was observed in unmarried patients, tumors located in other parts of the breast, IDC, moderately differentiated tumors, BCS and 1 to 3 postoperative lymph node positive subgroups.

## Discussion

Our results show that for breast cancer patients with cT_1-2_N_1_M_0_ who received NAC, postoperative RT significantly improved the patients’ OS, but no significant difference was found in the improvement of CSS. It is worth noting that the results of subgroup analysis of patients with right-sided breast cancer showed that postoperative RT had a positive impact on OS and CSS. This result may be related to the lower risk of severe cardiotoxicity faced by patients with right-sided breast cancer when receiving postoperative RT relative to patients with left-sided breast cancer [10]. Long-term follow-up studies have found that RT-induced cardiotoxicity may increase breast cancer treatment-related mortality, partially offsetting the survival benefits of RT [11, 12]. Due to the anatomical location, the radiation dose received by the heart during RT for right-sided breast cancer is significantly reduced compared with left-sided breast cancer, thereby significantly reducing the risk of death caused by cardiotoxicity [11].

The benefit of postoperative RT for patients with cT_1 − 2_N_1_M_0_ breast cancer who receive NAC has been controversial. A retrospective study based on the Japanese breast cancer registry [13] included 3,226 patients who underwent NAC and MAST (cT_1 − 4_N_0−2_M_0_), and found that for ypN_0 − 1_ patients, postoperative RT did not significantly benefit in terms of LRR and OS. Another retrospective study found that in patients with cT_1 − 2_N_1_M_0_ breast cancer who received NAC, whether ypN_0_ or ypN_+_, postoperative RT could improve OS [14]. However, other studies have shown that omitting RT does not increase the risk of local recurrence or death in patients with stage II-III ypN_0_ breast cancer who undergo MAST after NAC [15, 16]. A meta analysis also confirmed that postoperative RT did not improve disease-free survival (DFS) and OS in ypN_0_ patients who underwent MAST after NAC treatment [17].

Our results are similar. The survival benefits of RT were not observed in the ypN_0_ subgroup, but RT significantly improved OS in the ypN_+_ subgroup. In addition, we also observed the benefits of OS in the BCS subgroup. The postoperative recurrence rate of BCS is high, even with postoperative RT, the LRR is still higher than that of MAST without RT [18]. Postoperative RT can reduce the risk of local recurrence by 2/3 and mortality by about 1/6 [19]. In the RAPCHEM study [9], it was found that among patients in the ypN_0_ low-risk group who did not receive RT (60% received BCS), the 5-year LRR and OS were 2.1% and 95.5%, respectively, so RT was considered to be omitted. Other studies have also shown that postoperative RT is not associated with local recurrence in patients with ypN_0_, regardless of whether they undergo BCS or MAST [20]. The joint analysis of NSABPB-18 and B-27 [21] supported the necessity of RT in patients with ypN_+_, especially in patients with BCS, and showed a significant decrease in the local and regional lymph node recurrence rate of breast cancer in this group in the past 10 years.

At the same time, the key question is whether sentinel lymph node biopsy (SLNB) can accurately evaluate the axillary status after NAC, which may avoid axillary RT or axillary lymph node dissection (ALND). According to the NSABP-B32 study [22], for patients with clinically diagnosed axillary lymph node negative (cN_0_), there is no significant difference between SLNB and ALND in OS and DFS, and can effectively reduce postoperative complications. Although the successful detection rate of SLNB decreased slightly in patients with pre-NAC cN_0_ (from 98–95%) [23], the false negative rate of 7% was still lower than the acceptable standard of 10% [24]. For patients with cN_2_, it is generally recommended that ALND be used directly, regardless of whether there is a downgrade after NAC. However, whether patients with cN_1_ should undergo SLNB after NAC is still controversial.

Studies have shown that about 41% of cN_1_ patients achieve axillary pCR after NAC [25]. These patients receiving axillary RT or ALND have no significant benefits, but may increase the risk of treatment complications. However, Z1071 [25] and SENTINA [26] studies revealed that the detection rate of SLNB in cN_1_ patients after NAC was 92.8%, and the false negative rate was 12.6%, suggesting that the false negative rate was high. In a study of 243 breast cancer patients with cT_1 − 3_N_1_M_0_, it was found that the strategy of using double-dye tracing and SLNB to detect more than 3 negative sentinel lymph nodes effectively avoided ALND and axillary RT, and the axillary recurrence rate was only 0.4% in the group noRT [27]. This shows that by using dual dye tracing method, increasing the number of sentinel lymph node detection, combined with targeted axillary dissection (TAD) and imaging evaluation, the false negative rate can be significantly reduced and the accuracy of SLNB can be enhanced.

However, our study has several important limitations. First, since our study was retrospective, selection bias still existed even though PSM was performed to reduce it. Secondly, despite our large sample size, the occurrence of death events is insufficient, which may affect our statistical power. Finally, in the SEER database, it lacks information on specific chemotherapy regimens, RT doses, specific regions, and other clinical risk factors such as ki67 and BRCA1 and BRCA2-related mutations, which may affect the reliability of our results.

## Conclusion

Studies based on SEER database show that the OS of cT_1 − 2_N_1_M_0_ breast cancer patients receiving NAC is improved after postoperative RT, but the CSS is not significantly increased. However, for the subgroup of patients with right-sided breast cancer, postoperative RT not only improved OS but also significantly improved CSS. These findings need to be further verified by more prospective clinical trials.

## Data Availability

Data from the SEER program is available for public. The data supporting the conclusions of this article are available in the Surveillance Epidemiology, and End Results (SEER) database (https://seer.cancer.gov/).

## Abbreviations

NAC: neoadjuvant chemotherapy
SEER: Surveillance, Epidemiology, and End Results
PSM: propensity score matching
OS: overall survival
CSS: cancer-specific survival
DFS: disease-free survival
LRR: local recurrence rate
IDC: invasive ductal carcinoma
ILC: invasive lobular carcinoma
IDLC: invasive ductal and lobular carcinoma
BCS: breast-conserving surgery
ALND: axillary lymph node dissection
TAD: targeted axillary dissection
RT: radiotherapy
noRT: non-radiotherapy
pCR: pathological complete response rate
RMST: restricted mean survival time
MAST: mastectomy
SLNB: sentinel lymph node biopsy
